# Estimating a Sleep Apnea Hypopnea Index Based on the ERB Correlation Dimension of Snore Sounds

**DOI:** 10.3389/fdgth.2020.613725

**Published:** 2021-02-01

**Authors:** Limin Hou, Qiang Pan, Hongliang Yi, Dan Shi, Xiaoyu Shi, Shankai Yin

**Affiliations:** ^1^School of Communication and Information Engineering, Shanghai University, Shanghai, China; ^2^Department of Otolaryngology, Shanghai Jiao Tong University Affiliated Sixth People's Hospital, Shanghai, China

**Keywords:** apnea hypopnea index, correlation dimension, non-linear acoustic characteristics, snore sound, sleep apnea hypopnea syndrome

## Abstract

This paper proposes a new perspective of analyzing non-linear acoustic characteristics of the snore sounds. According to the ERB (Equivalent Rectangular Bandwidth) scale used in psychoacoustics, the ERB correlation dimension (ECD) of the snore sound was computed to feature different severity levels of sleep apnea hypopnea syndrome (SAHS). For the training group of 93 subjects, snore episodes were manually segmented and the ECD parameters of the snores were extracted, which established the gaussian mixture models (GMM). The nocturnal snore sound of the testing group of another 120 subjects was tested to detect SAHS snores, thus estimating the apnea hypopnea index (AHI), which is called AHI_ECD_. Compared to the AHI_PSG_ value of the gold standard polysomnography (PSG) diagnosis, the estimated AHI_ECD_ achieved an accuracy of 87.5% in diagnosis the SAHS severity levels. The results suggest that the ECD vectors can be effective parameters for screening SAHS.

## Introduction

Snoring is one of the most important symptoms of Sleep Apnea Hypopnea Syndrome (SAHS) and carries much information for diagnosing the upper airway disorder (1). Snoring sounds can be recorded by a non-contact microphone using acoustical property analysis for the screening of SAHS (2, 3). The pitch and spectral characteristics of snoring have been widely applied (4, 5). The total airway response for a snore was extracted to examine SAHS by a higher-order statistics algorithm (6). Multiclass classification of snoring was acquired on the acoustic analysis of snore sounds (7). A genetic algorithm was applied to select the better features that can be extracted from the time and spectral domains of full-night recordings to determine the Apnea Hypopnea Index (AHI) value (8). The rhythmic variations in the snores were described to assess the AHI (9). Hidden Markov models with Mel frequency cepstral coefficients (MFCC) were used to classify subjects into different ranges of the AHI (10). Our previous work used snore spectral information to estimate the AHI (11). Traditional time and frequency analysis and the classic method for snore sounds were adopted in the studies mentioned above.

However, the irregular and turbulent airflow that is produced within the upper airway tissue vibrations that cause the snore, such as the intensity of respiratory airflow, vibration on the soft palate, thick tongue root, and epiglottic hypertrophy, etc. could be non-linear (12). It was suggested that linear analysis methods were limited and that more useful information could be obtained using chaos theory to analysis the snore (13, 14). The largest Lyapunov exponent and entropy were calculated to classify snore-related sounds with multiclass system (15).

In this paper, a new correlation dimension was proposed to analyze the non-linear properties of snoring sounds for automatic AHI prediction. In contrast to the conventional correlation dimension, the all frequency region was divided into multi-sub-bands on an equivalent rectangular bandwidth (ERB) scale, and the correlation dimensions were calculated in each sub-band. Therefore, ERB correlation dimension (ECD) vectors were extracted rather than a single correlation dimension. The gaussian mixture model (GMM) was applied to build the ECD vector models. Whole-night snore sounds of patients were detected in our experiments, and then, the AHI_ECD_ values were estimated. The early experimental studies have been published in Chinese journals, when the experiment is the number of 60 snorers in reference (16). This research continues to now increased to 120 snorers. In other words, the experimental results of adding 60 people dropped slightly from the original 90 to 87%. It illustrates the robustness of new features. This study further adds a comparison with the classic feature MFCC. Compared to the polysomnography (PSG) diagnosis, our non-linear features achieved higher accuracy than the MFCC based snore spectrum information in the severity levels of the SAHS. The ECD vectors were found to characterize various severity levels of snores.

## ERB Correlation Dimension

The phase space reconstruction technique has been widely used in the field of chaos and fractal theory (17), and it has been used in some applications in medical and speech signals (18–20). It could be more comprehensive disclosure of snore implied information by transforming them to high-dimensional space. The general representation of a snore is a time series. Let a one-dimensional discrete series *s*(*n*) be denoted by the snore signal, that is get by sampling rate *Fs*.

Based on the Takens embedding theorem (21), the phase space reconstruction could transform a one-dimensional time series into a high-dimensional phase space vector *Y* ϵ *R*^*m*^ as in Equation (1).


$$\begin{array}{l} Y=\left[Y_{1}\;Y_{2}\;\cdots \;Y_{i}\;\cdots \;Y_{I}\right] \end{array}$$

Where


(1) $$\begin{array}{r} Y_{i}={\left[s\left(n_{i}\right)\;s\left(n_{i}+\tau \right)\;\cdots \;s\left(n_{i}+\left(m-1\right)\tau \right)\right]}^{T} \end{array}$$

Here, τ is the time delay, and m is the embedding dimension. The reconstruction vector *Y* is an m-dimensional vector with *I* phase points. The appropriate time delay was selected according to the autocorrelative function (AR function) (22). The time delay τ is an integer multiple of the sampling interval: τ =*n/Fs*.

The purpose of choosing the embedding dimension is to make the original chaos attractor and the reconstructed attractor topology equivalent. We used the false nearest neighbor (FNN) method to determine the embedding dimension *m* (23). As the embedding dimension *m* increases, the orbit of the chaotic motion will gradually open, and the false nearest neighbors will be gradually eliminated, until the trajectory tends to be stable and the proper *m* is obtained (24). When the frame length was > 150 ms, the slope of the correlation integral curve increased very slowly. Finally in our snore work, the time delay of 0.75 ms and the embedding dimension of 15 were confirmed by the above method with a frame length of 150 ms.

**Figure 1:**
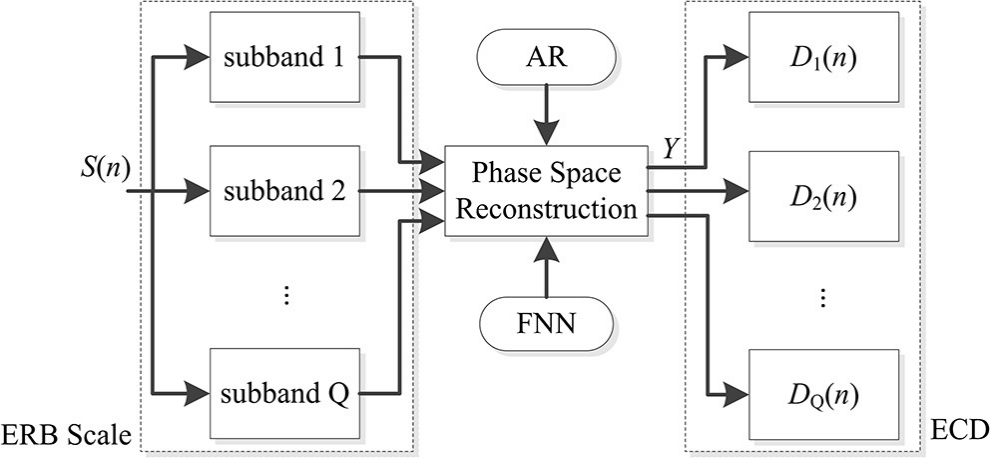
Flow chart of the ECD calculation.

The traditional correlation dimension has only a single parameter, it is difficult to make a more comprehensive analysis of complicated signals. Based on the ERB scale related to auditory perception (25, 26), several sub-bands were divided from the whole frequency band of the snore signal. Phase space reconstruction was performed in each of the sub-band signals of the snore. Then, the correlation dimension on these sub-bands were calculated, which obtained auditory sub-band ERB correlation dimension (ECD) vectors. The flow chart of extracting the ECD is shown in Figure 1.

Equation (2) is the correlation integral *C*_*q*_ (*I, r*) of the qth subband, which calculates the probability that the distance of paired (*Y*_*iq*_, *Y*_*jq*_) is smaller than *r*.


(2) $$\begin{array}{r} C_{q}\left(I,r\right)=\frac{1}{I\left(I-1\right)}\overset{I}{\underset{i,j=1}{\sum }}\theta \left(r-|Y_{i,q}-Y_{j,q}|\right) \end{array}$$

Where θ (·) is the Heaviside function, and if x < 0, θ (x) = 0; if x > 0, then θ (x) = 1. The correlation dimension *D* is estimated based on the ratio of the logarithm of the correlation integral and the logarithm of the distance *r*, as in Equation (3).


(3) $$D_{q}=\frac{\ln C_{q}(I,r+\Delta r)-\ln C_{q}(I,r)}{\ln\left(r+\Delta r\right)-\ln r}$$

Therefore, the correlation dimension of the qth subband was calculated by Equations (2, 3) based on the Grassberger-Procaccia (GP) algorithm (24). Finally, we get the ECD vector by arranging and integrating ERB subband' correlation dimension, as in Equation (4).


(4) $$\begin{array}{r} \text{EC}\text{D}\left(n\right)=\left[D_{1}\left(n\right)\;D_{2}\left(n\right)\;\cdots \;D_{q}\left(n\right)\;\cdots \;D_{Q}\left(n\right)\right] \end{array}$$

In this study, the gap of the adjacent sub-band was one bandwidth of the ERB scale, the frequency range of 60 Hz to 4 kHz was divided into 24 sub-bands, that is *Q* = 24, and the 24-dimensional vector of the auditory sub-band ECDs were extracted.

Moore and Glasberg proposed the relationship between frequency and ERB scale (25, 26), as in Equation (5).


$$\begin{array}{l} ERB\left(f\right)=6.23f^{2}+93.39f+28.52 \end{array}$$

(5) $$\begin{array}{r} ERBS\left(f\right)=11.17628*\text{ln}\left(1+\frac{46.06538f}{f\;+\;14678.49}\right) \end{array}$$

where *f* is physical frequency in kHz. ERB(*f*) is the calculated rectangular bandwidth of the equivalent filter in Hz. ERBS(*f*) is the ERB scale in physical frequency *f* in Hz.

## Snore ECD Features

### Snore Data

Snore sounds were recorded in the sleep monitoring laboratory in the Department of Otolaryngology of Shanghai Jiao Tong University Affiliated Sixth People's Hospital by a non-contact ambient microphone, and simultaneously, polysomnography (PSG) diagnosis was performed. The recording uses a non-contact microphone Sony EM-C10, which is hung on the head of the bed, about 30 cm away from the patient's nose and mouth. The recording sound card is Creative Audigy 4 Value, the desktop computer is Dell Inspiration 570, the recording software Adobe Audition 3.0, the sampling frequency is 8 kHz sampling, 16 bit quantization, and saved as WAV audio files. The recording duration is 7 h from 10:30 p.m. to 5:30 a.m. the next morning. In this test experiment, the half hour before the beginning and the end are removed, and 6 h of recording are used. The details of recording for snore sounds were the same as literature (11).

The AHI_PSG_ was the apnea hypopnea index as diagnosed by the gold standard PSG. The severity levels of SAHS were determined using the AHI value. The subjects with AHI > 30, 15 < AHI ≤ 30, 5 ≤ AHI ≤ 15, and AHI < 5 were classified as severe (S), moderate (M), mild (L) SAHS, and non-SAHS (N), respectively (27).

**Figure 2:**
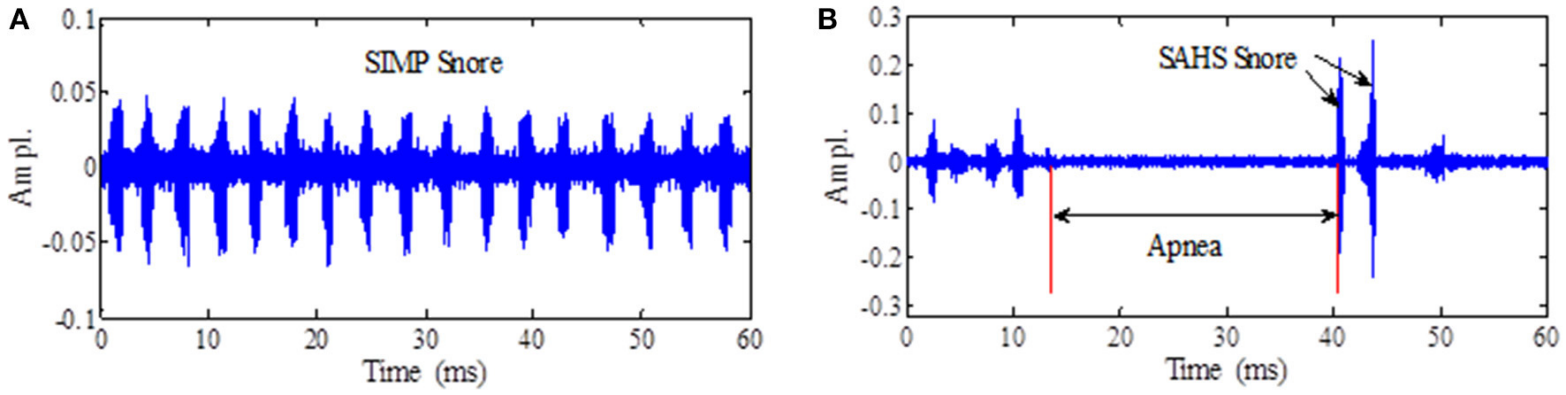
**(A)** The simple snore wave. **(B)** The SAHS snore wave.

The 213 subjects were consecutively recruited. In our experiment training phase, the snore episode was cut artificially from the sound of overnight recordings by a non-contact microphone on the bedhead, which included 93 subjects from 213. According to synchronized PSG nocturnal monitoring data, there were two types of snore episodes that were labeled. One was snoring sound labeled snore events by PSG diagnosis, which was only resounding and occurred periodically. The other was a loudly snoring sound appearance behind apnea or hypopnea events labeled by PSG. We called the former a simple snore (SIMP) and the latter a SAHS snore (SAHS). These are shown in Figure 2.

Another 120 subjects from 213 were as a test data set by their overnight recording of sounds. We removed the starting 30 min and the ending 30 min of recording. The remaining 6 h audio signal (11) were used for our test experiments as shown in Table 1.

**Table 1:** Snore data for training and test.

	**Training Data**			
	**N**	**L**	**M**	**S**
Subjects (number)	10	23	24	36
Gender (M/F)	9/1	21/2	23/1	36/0
Age (years)	42.1 ± 8.5	46.2 ± 12.4	40.4 ± 13.2	45.6 ± 12.5
AHI_PSG_ (events/h)	2.4 ± 1.4	10.8 ± 3.5	24.5 ± 3.8	57.0 ± 16.8
SIMP Episodes (number)	339	919	480	430
SAHS Episodes (number)	55	376	480	916
	**Test Data**			
Subjects (number)	30	30	30	30
Gender (M/F)	19/11	26/4	25/5	27/3
Age (years)	29.9 ± 8.6	40.7 ± 12.4	43.0 ± 13.8	38.9 ± 11.7
AHI_PSG_ (events/h)	1.9 ± 1.6	9.3 ± 3.0	22.2 ± 4.3	62.5 ± 17.8
Recording length (minutes)	360 × 30	360 × 30	360 × 30	360 × 30

### The Largest Lyapunov Exponent

**Figure 3:**
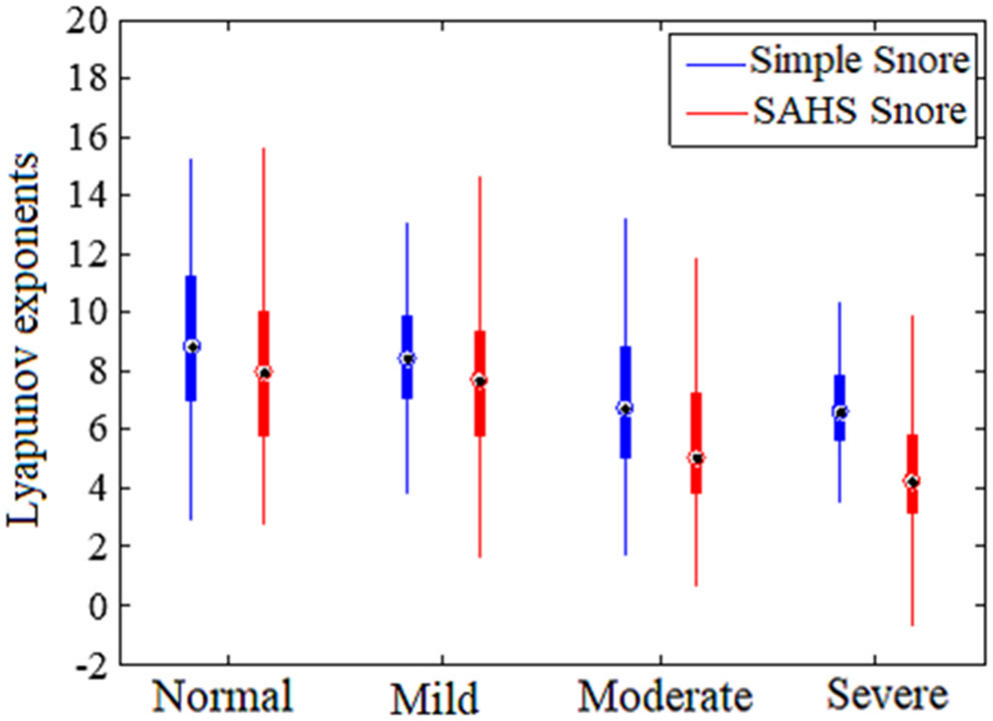
Largest Lyapunov exponents of snore for different SAHS severity levels.

The largest Lyapunov exponent (LLE) of snores has been calculated to measure the rate of local divergence of nearby trajectories in the state space from dynamical systems theory (28). The LLE of all type snores are shown in Figure 3. The LLE of the four types of simple snore and SAHS snore are all positive. A few of the severe types have the Lyapunov exponent of SAHS snore < 0, accounting for only 2.4% of the severe type of snoring episodes. The LLE of other types of snores did not appear negative, which also shows that the chaos of snoring is universal. This conclusion is consistent with other researches (13, 15, 29).

The mean of the LLE distribution of simple snore is greater than the LLE distribution of SAHS snore in same severity level. This phenomenon is common in the four types of snore signals. In moderate and severe levels, the difference between the two means of SIMP snore and SAHS snore is increasing. The results reveal that the orbital divergence speed of SIMP snore is greater than that of the SAHS snore, and is consistent with the other study (29). The LLE distribution suggests that unconscious airflow from simple snoring may have more freedom to roam, while SAHS snoring may form a certain trend of airflow after being squeezed in the narrow upper airway.

### ECD Calculation

**Figure 4:**
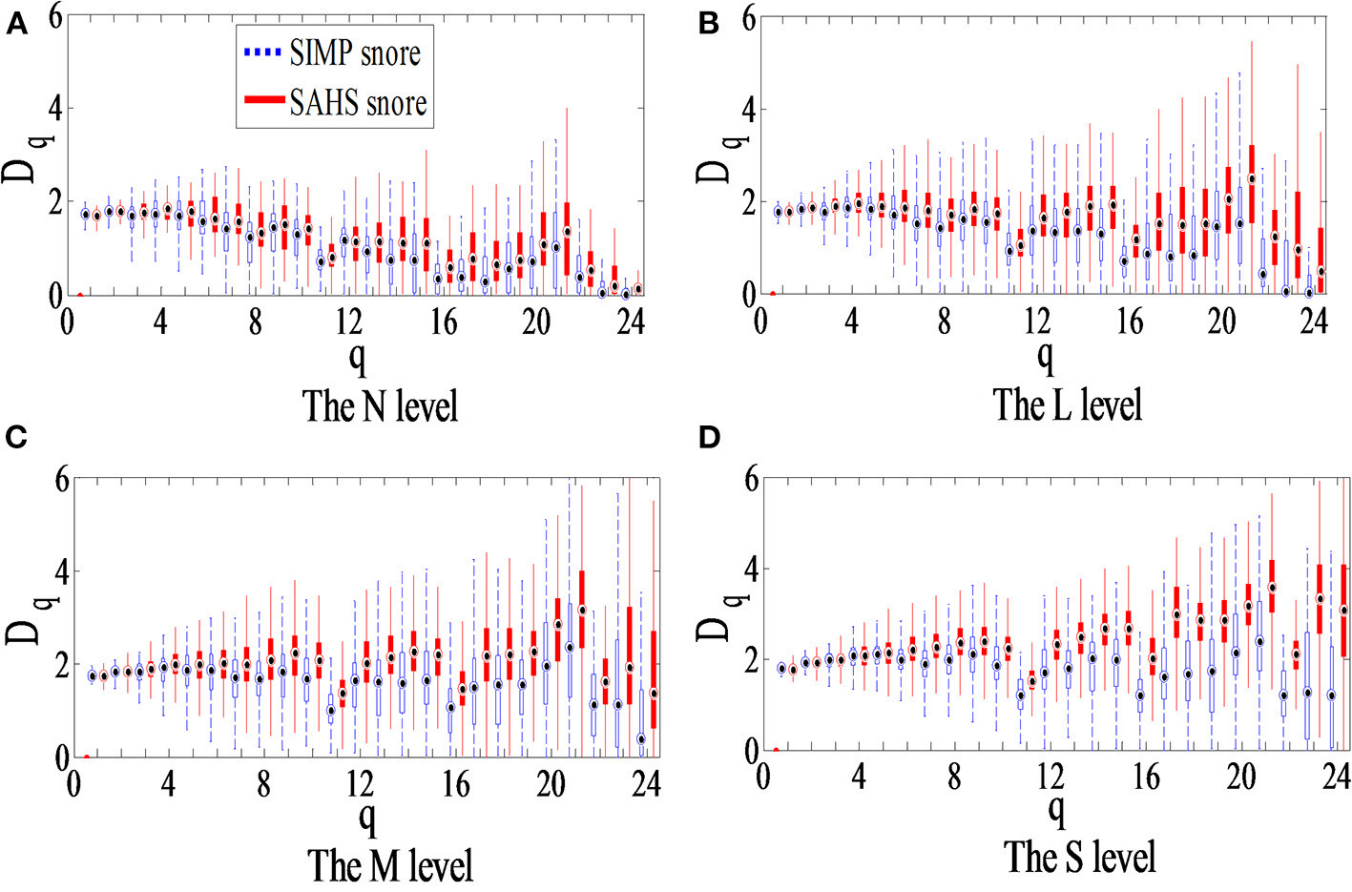
Box plot of ECD vector for different SAHS severity levels. **(A)** The N level, **(B)** The L level, **(C)** The M level, and **(D)** The S level.

According to the illustration in Figure 1, the ECD of the snore from the training data in Table 1 were calculated. The distribution of the ECD vectors in each sub-band of the SIMP snore and SAHS snore of the N, L, M, and S levels, respectively, are shown in Figures 4A–D. The ECD vectors distinctively increased with the aggravation of the SAHS severity level in the middle and high-frequency sub-bands. Moreover, the distributions of the ECD vectors were not exactly the same for the SIMP snores and the SAHS snores at the same severity level, and the ECDs of the SAHS snores were always higher than those of the SIMP snores.

**Figure 5:**
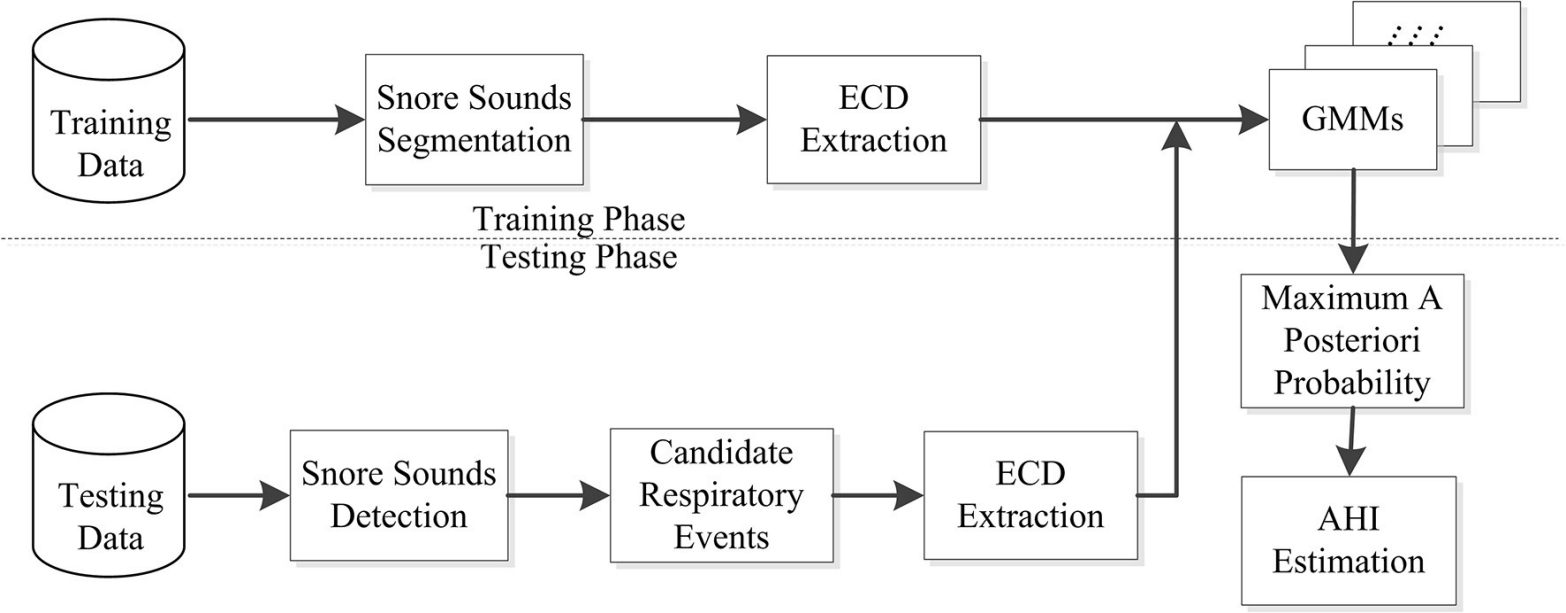
Flow chart of the snore training and testing system.

In our study, the SIMP and SAHS snores of four levels (N, L, M, and S) were modeled using the Gaussian Mixture Model (GMM), which formed eight types, including N-simp, N-sahs, L-simp, L-sahs, M-simp, M-sahs, S-simp, and N-sahs. The ECDs of the training data in Table 1 were extracted to model eight GMMs for the training phase (30), and are showed in Figure 5.

## Results and Discussion

### Results

**Figure 6:**
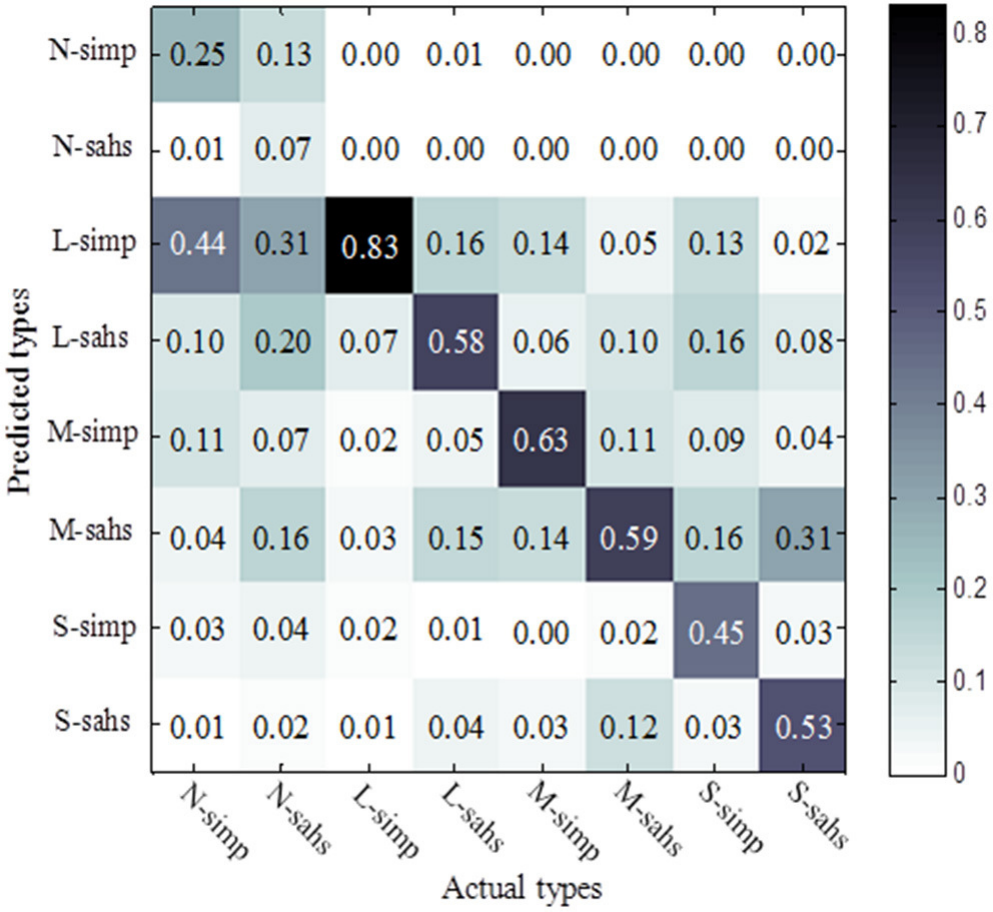
Confusion matrix of eight types.

Mixture Number of GMM were assigned 2, 12, 12, and 8 for both SIMP and SAHS snore of N, L, M, and S level, respectively. GMM was solved by expectation-maximization (EM) algorithm. Two-fold cross-validation method was employed to evaluate the performance of clustering and classification of GMM regarding training data in Table 1. For each type of snore, the rate of being classified as different types is shown in Figure 6.

According to the PSG clinical diagnosis definition, AHI is the number of respiration events per hour of sleep. The ECD-calculated AHI is AHI_ECD_ as in Equation (6).


(6) $$AHI_{ECD}=\frac{Number\;of\;sleep\;respiratory\;events}{Duration\;of\;nocturnal\;sleep}\;\;\;\;\;\;events/h$$

Figure 5 shows the testing phase, there were another 120 participants for the testing data, which consisted of 30 subjects for each severity level among N, L, M, and S in Table 1. Firstly, automatic endpoint detection was performed for snore signals of whole-night recordings to detect the snore sounds (16, 31). Thus, we obtained candidate respiratory events based on the unique rhythm of snores (16, 31). Then, the ECD vectors of these candidate respiratory events were extracted, and we calculated the probabilities of matching with eight GMMs. On the basis of the Bayesian maximum posterior probability rule, the maximum posterior probability winner among the eight GMMs was the snore type. When some snore episodes in a candidate respiratory event were classified as any SAHS snores among N, L, M, and S levels by the GMM, that candidate respiratory event was a true sleep respiratory event. Finally, the AHI_ECD_ score was estimated by the number of sleep respiratory events and the nocturnal sleep duration, as in Equation (6).

In the same way, we extracted another feature set that is MFCC, and estimated the AHI_MFCC_ score also. The MFCC is a classical feature and widely used automatic speech recognition. All experiment results in precision and recall were listed in Table 2.

**Table 2:** Precision and recall of AHI_MFCC_ and AHI_ECD_ compared AHI_PSG_.

**Levels**	**N**	**L**	**M**	**S**	**Total Correct**	**Mean**
Subjects(number)	30	30	30	30	120	
Precision of AHI_MFCC_	85.71%	64.28%	70.37%	96.66%	96	79.25%
Recall of AHI_MFCC_	100%	60%	63.33%	96.66%	96	80.00%
Precision of AHI_ECD_	100%	81.48%	75.75%	93.75%	105	87.74%
Recall of AHI_ECD_	93.33%	73.33%	83.33%	100%	105	87.50%

Comparisons of AHI_ECD_, AHI_MFCC_, and AHI_PSG_ values of each subject, consistency with the severity of the gold standard PSG diagnosis was correctly screened. There are 120 testers in Table 2, including 30 people of four different severity levels. As a result of the MFCC classic feature test, 30 people who are non-ASHS can correctly estimate. Twelve of the 30 mild patients were incorrectly classified as non-SAHS or moderate SAHS types. Eleven of 30 moderate patients were wrongly assorted as mild or severe SAHS types. Thirty patients with severe patients, one of whom were mistakenly estimated as moderate SAHS patients. Compared to AHI_PSG_ in the diagnosis of the SAHS severity level, and the AHI_MFCC_ estimation achieved the mean precision and recall of 79.25 and 80.00%, respectively, as shown in Table 2.

As a result of the ECD feature test, two out of 30 non-SAHS people were mistakenly estimated to be mild patients. Thirty patients with mild patients, eight of whom were incorrectly estimated to be non-SAHS or moderate patients. Of the 30 patients with moderate disease, five of them were incorrectly estimated to be mild or severe SAHS patients. Thirty people with severe patients were correctly estimated. The AHI_ECD_ estimation using our proposed method achieved, respectively, the mean precision and recall of 87.74 and 87.50% compared to AHI_PSG_ in the diagnosis of the SAHS severity level as shown in Table 2.

The precision and recall of AHI_ECD_ are higher than AHI_MFCC_ in mild and moderate levels especially.

Comparisons results of AHI_ECD_ and AHI_MFCC_, both features are good at both ends (i.e., non-SAHS and severe patients). However, for patients with mild SAHS and moderate SAHS, the number of errors by using MFCC is higher than ECD feature. The precision and recall of AHI_ECD_ are higher than AHI_MFCC_ in mild and moderate level especially. New fractal features achieve better results than classical spectral features. Relative literature (16), this work increased the number of patients in test experiments from 60 to 120 and adds to compares them with the classic feature MFCC. Therefore, the experimental results of this paper are almost the same as the initial experiments, once again confirming the advantages of the new features.

### Discussion

Most of the previous studies of detecting snore used the acoustic characteristics of the speech signal of the active pronunciation (4–11), that is limit. Because the snoring contained more breathing sounds than speech. This airflow has more randomness and is generated by passive vocalization. Thereby, we proposed a new feature from the chaos and fractal theory to characterize the irregular extent of snore.

**Figure 7:**
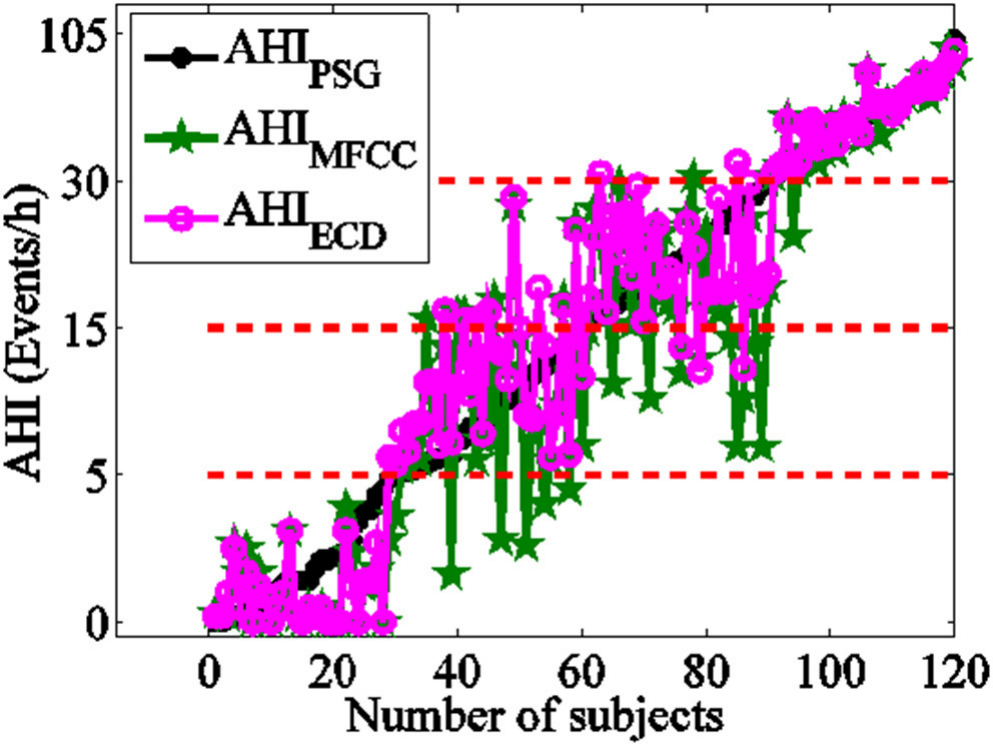
The compared between AHI_ECD_, AHI_MFCC_, and AHI_PSG_.

The AHI_ECD_ by new features is closer to clinical diagnosis results than AHI_MFCC_ by conventional parameters. The distribution scatter plot of AHI_PSG_ vs. AHI_MFCC_, AHI_ECD_ is shown in Figure 7. The black asterisk represents the result of PSG diagnosis AHI_PSG_, the green pentagram represents AHI_MFCC_, and the purple circle represents AHI_ECD_, and the red dotted line represents the boundary of different severity. The cohen's kappa coefficient of AHI_ECD_ and AHI_PSG_ consistency is 0.833, and AHI_MFCC_ and AHI_PSG_ consistency is 0.733.

**Figure 8:**
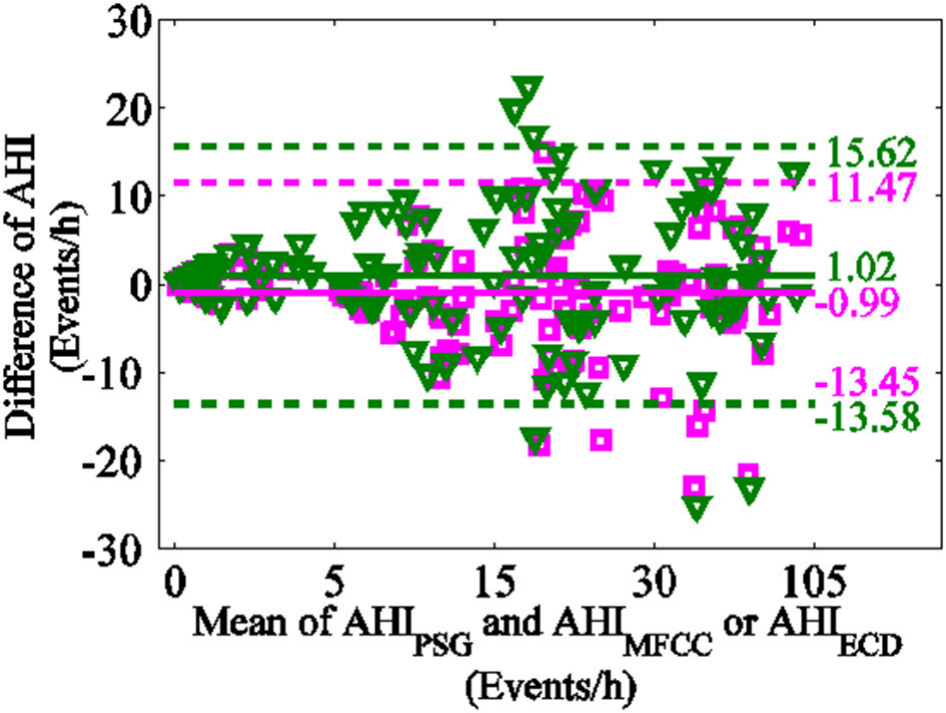
Bland-Altman-plot (the difference of AHI_PSG_ and AHI_ECD_ with pinkish, the difference of AHI_PSG_ and AHI_MFCC_ with green).

The Bland-Altman-plot is depicted in Figure 8. The ordinate represents the difference of AHI_PSG_ and AHI_ECD_ with pinkish, the difference of AHI_PSG_ and AHI_MFCC_ with green. The mean and variance of difference of AHI_ECD_ and AHI_PSG_ were smaller than AHI_MFCC_. Compared with PSG, 92.50% (111/120) of AHI_ECD_ falls within the consistency limit of 1.96 times variance, higher than 88.33% (106/120) of AHI_MFCC_. This further suggests AHI_ECD_ estimated by ERB correlation dimensions is more accordance with AHI_PSG_ than AHI_MFCC_.

In terms of the severity of SAHS, especially the N-type and the severe type, their frequency spectrum has obvious differences. Therefore, the MFCC parameters maintain a good performance for the judgment of these two types. The ECD feature is the same. However, for the intermediate types of mild and moderate, the accuracy of MFCC's outcome drops sharply. The ECD we proposed is much better than MFCC in these two types.

This paper presents a method to measure the degree of disorder of the snoring signal like noise, which were new features called the ECD vectors. The correlation dimensions of the high frequency sub-bands were larger than those of the low-frequency sub-bands in ECD vector. The maximal correlation dimension appeared in the 21st ERB sub-band as shown in Figure 2. This finding suggested that the SAHS snores contain much more irregular and fast-changing components in the high frequency range. The ECD vectors could reveal information that is consistent with the characteristic of the time-domain waveform of the snore. When the upper airway is blocked, the airway becomes shorter. When the upper airway rushes open, the airflow in the narrow area is squeezed, and the turbulent airflow is released. However, the snore spectrum is attenuated to a smaller magnitude in the high frequency range, and thus, it is difficult to give an appropriate description for the mild and moderate level of a snore, which could be to too small to distinguish different level. These non-linear methods are expected to provide useful information for better understanding of irregular snoring sounds (13, 14). MFCC includes only magnitude of snore spectral, but our ECD feature completes information in snore sound. When the upper airway is obstructed, the shortening of the airway leads to an increase in the medium and high frequency components, the airflow in the narrow area is squeezed, and some the rapid change component increases. The Fourier transform shows a characteristic of global decline and local prominence. Compared with MFCC, the ERB enlarged partially and highlighted the anomaly of the mid and high frequency components.

Inspired by the non-linear frequency scale and MFCC characteristics of the Mel spectrum, we use ERB to set the sub-band frequency interval to 8, 4, 2, and 1 ERB bandwidth, so that 3, 6, 12, and 24 subbands are obtained severally in formula (5). The obvious differentiation the snoring sounds of different severity appears when dividing three sub-bands but the details are not enough to distinguish well. As the number of subbands increases, more and more details provide a richer diversity of different severity level of SAHS. According to the distribution of the auditory filter, as it is divided into about 20 subbands in 4 kHz, a set of features is more effective. We adopted one ERB bandwidth and 24 subbands are obtained in formula (5).

No matter how many take the ERB scale, ECD features exhibits SAHS severity is directly proportional to the relationship, that is, the more severe the SAHS, the faster the ECD rises in the middle and high frequency regions, shown as in Figure 4.

However, the calculation of the correlation dimension was time-consuming. This limitation requires us to optimize the algorithm for the correlation dimension. The nature of the correlation dimension on the number of more subbands may need further study.

## Conclusions

Based on the previous experiment, we prove the chaotic nature of snoring sound by the LLE and perfect a new method for estimating the AHI value of SAHS using the correlation dimension vector for snore sounds, which was superior to the conventional spectrum analysis. The ECD vectors might be closely related to the SAHS severity level and reveal the effect of different SAHS severities on the upper airway. The correlation dimension of the sub-bands reveals the inherent information of the mid and high frequencies, while the Fourier transform has its limitations. Chaos provides many quantitative parameters for exploring the nature of this internal information. It could be a study about correlation between fractal dimension and internal physical properties of sleep respiratory sound. There is a positive effect on the development of a medical supplementary diagnosis and in-home healthcare in the internet era.

## Data Availability Statement

The original contributions presented in the study are included in the article/supplementary materials, further inquiries can be directed to the corresponding author/s.

## Ethics Statement

The studies involving human participants were reviewed and approved by Department of Otolaryngology, Shanghai Jiao Tong University Affiliated Sixth People's Hospital. Written informed consent for participation was not required for this study in accordance with the national legislation and the institutional requirements.

## Author Contributions

All the co-authors contributed to this work. LH was responsible for all scheme design and about correlation dimension algorithm work of this paper. QP organized the writing work of this paper and MFCC programming work. HY and SY participated on the clinical diagnosis by PSG and actively discussed in this work. DS programmed the EM algorithm work of this paper. XS programmed the result analysis work of this paper.

## Conflict of Interest

The authors declare that the research was conducted in the absence of any commercial or financial relationships that could be construed as a potential conflict of interest.
